# Social support during childbirth as a catalyst for early breastfeeding initiation for first-time Nigerian mothers

**DOI:** 10.1186/1746-4358-4-16

**Published:** 2009-12-10

**Authors:** Imran O Morhason-Bello, Babatunde O Adedokun, Oladosu A Ojengbede

**Affiliations:** 1 Department of Obstetrics and Gynaecology, College of Medicine, University College Hospital, Ibadan, Oyo State, Nigeria; 2 Department of Epidemiology, Medical Statistics, and Environmental, Health, College of Medicine, Ibadan, Oyo State, Nigeria

## Abstract

**Background:**

Initiation of breastfeeding can be difficult in a busy maternity centre with inadequate manpower and social support. This study aims to explore the role of psychosocial support offered by companions on breastfeeding initiation among first-time mothers.

**Methods:**

This is a secondary data analysis of a randomised controlled trial conducted among women attending the antenatal clinic of the University College Hospital, Ibadan, Nigeria in 2007. Those in the experimental group were asked to bring someone of their choice to the labour room to act as a companion; the comparison group received standard care. The results of 209 HIV negative women who had vaginal births were analysed. The main outcome measure was time to initiation of breastfeeding after childbirth.

**Results:**

Of the total, 94 had companions during labour while 115 did not have a companion. The median time to breastfeeding initiation was significantly shorter in those with companions compared to controls (16 vs. 54 minutes; p < 0.01). The cumulative survival analysis indicated that all in the treatment group had initiated breastfeeding by 26 minutes, while among the control group none had commenced at 30 minutes post-delivery with some as late as 12 hours. After Cox regression analysis was used to adjust for possible confounders, the outcome still showed a significant hazard ratio of 207.8 (95%CI 49.2, 878.0; p < 0.01) among women who were supported by a companion.

**Conclusion:**

Use of companions during labour is associated with earlier time to breastfeeding initiation among first-time mothers in Nigeria.

**Trial Registration:**

Australian New Zealand Clinical Trials Registry: ACTRN12609000994280.

## Background

Time to breastfeeding initiation is one of the commonly reported independent predictors of exclusive breastfeeding in many communities [1]. Over the years, UNICEF has promoted breastfeeding initiation within half an hour of childbirth as an important strategy to reduce perinatal and infant morbidities and mortality, and by extension to support the attainment of Millennium Development Goal 4: reduce child mortality [2,3]. Other predictors of breastfeeding initiation include educational level, parity, age, socioeconomic status and ethnicity [4,5].

According to the 2003 National Demographic Health Survey (NDHS) of Nigeria, breastfeeding initiation varies with many background characteristics [6]. In this survey, 40% of women, who delivered at a health care facility where there are professionals, initiated breastfeeding within an hour compared to less than 30% in those who delivered elsewhere. In addition, there is a considerable variation by region with the lowest rate in the Southwest - predominantly Yorubas (13%) to as high as 58% in the South East, which consists of the mainly Igbo ethnic group. The 25 percent of Hausa-Fulanis that are mainly from North-West and East of the country initiated breastfeeding early.

There are various sociocultural norms that act as barriers to the practice/adoption of exclusive breastfeeding (EBF) by mothers in Nigeria [7-9]. The cultural barrier against breastfeeding with colostrum has been previously reported in Nigeria, as the fluid is regarded as a "poison" [10]. In addition, use of prelacteal feeds to complement breastfeeding has also remained a challenge in Nigeria [10].

The Baby Friendly Initiative (BFI) was launched in Nigeria in the early 1990s with the sole aim of integrating all the Ten Steps into the health care system with the intention of promoting breastfeeding practices to all nursing mothers except those with HIV infection [11]. The fourth Step is to encourage initiation of breastfeeding within half an hour of childbirth [12]. Since then, the authority of the University College Hospital, Ibadan (a tertiary public health institution) had adopted this policy and pronounced the hospital as one of the BFI designated sites (as is the practice in Nigeria). In spite of this, the majority of studies that have evaluated breastfeeding practices in Nigeria have reported low levels of exclusive breastfeeding.

The decision of prospective mothers to initiate breastfeeding early is therefore crucial to the duration of EBF and future commitments. Several studies have shown that maternal intention, offering prenatal health education classes, knowledge of the partner's/husband's favourable disposition and family support at home positively influenced the decision to initiate breastfeeding [13-15]. Apart from these, events around the childbirth process such as mode of delivery, labour experience amongst others have also been noted to significantly influence both early initiation and duration of breastfeeding [5].

The presence of a companion who offered psychosocial support to women during childbirth has been reported to be associated with positive breastfeeding practices [16]. For example, Hofmeyr et al in 1991 reported that social support during labour had a significant positive impact on exclusive breastfeeding even among adolescent mothers at six weeks postpartum [17]. It is based on this finding from South Africa and many others, that a secondary data analysis of a randomised controlled trial conducted among Nigerian parturients [18] on the effect of psychosocial support during childbirth was conducted to explore the strategy of using companions during labour to promote early breastfeeding initiation among first-time mothers.

## Methods

The data used in the study are part of a larger one involving a randomised controlled trial of the effect of psychosocial support in labour [18]. The data of the primigravidae in each arm of the study were extracted for analysis.

The original study was conducted at the University College Hospital (UCH), an 850 bed tertiary health institution located in Ibadan, south west Nigeria. Ethical clearance was obtained from the Institutional Review Board of University of Ibadan and UCH ethics committee. Participants were recruited at the antenatal clinic (ANC), where an average of 120 pregnant women are seen per week. At each clinic, the routine antenatal visits involve registration, vital signs measurement and group health talk before individual consultation with doctors. Breastfeeding is usually included in the group health talks. Early initiation, exclusive breastfeeding, duration and other issues that will facilitate a positive attitude towards breastfeeding are emphasized.

The labour ward has an admission room, first stage, delivery suite and a theatre. Each of the five cubicles have two beds that are screened to ensure privacy. On every duty shift, there are two residents and at least two house surgeons with a consultant attending each day. Five midwives, an anaesthetist and a paediatrician are also on duty at any time.

In the labour ward, the routine includes initial assessment including history and clinical examination. Those in established labour are admitted for partographic monitoring until second stage after which they are transferred to the delivery room. Sometimes, intermittent opiod analgesia is administered at the clinician's discretion. Relations of patients are usually barred from the labour suite.

Women with an anticipated vaginal birth were enrolled and allocated to either the experimental or control group using a randomisation sequence between 30 to 32 weeks after informing them in detail about the study during ANC. All were informed that their participation was entirely voluntary. A written consent was then obtained from each participant. Those in the experimental group were asked to bring someone of their choice to the labour room to act as a companion. The experimental group consisted of women who received routine care and social support while the control group received only routine care. The two groups of women were monitored for clinical outcomes from the onset of the diagnosis of labour until two hours after delivery. The exclusion criteria included: cervical dilatation of > 6 cm; contraindication to vaginal delivery; previous caesarean section; intrauterine fetal death; planned induction; multiple pregnancy; malpresentation; and chronic medical disorders.

**Figure 1:**
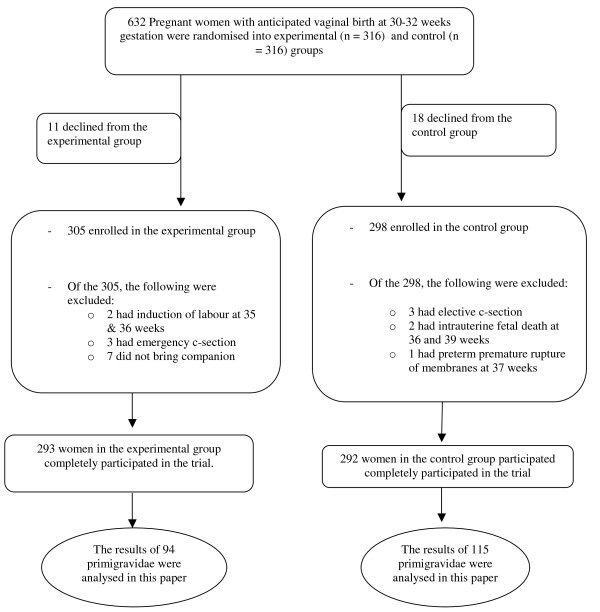
**The participant flow in the trial**.

The minimum sample size was calculated with an intention to detect a minimum difference of 10 percentage points in caesarean section rate (the major outcome for the study) between the groups at 80% power and 5% level of significance. The current caesarean section rate in UCH was 28.9% [19] and this was used for sample size calculation which gave a sample size of 275. The number of women analysed in the experimental and control group eventually were 293 and 292 respectively (Figure 1).

The randomisation sequence was generated by a statistician using a table of random numbers. Random permuted blocks of size 4 were used to ensure a balanced design. The blocks are the possible permutations of a sequence of four allocations - two women each belonging to the experimental and control groups. There are 6 possible arrangements of this sequence (ABAB, ABBA, BABA, BBAA, BAAB, AABB) - where A and B represent the groups. On the table, numbers 1 to 6 were assigned to each arrangement and those outside this range were excluded. Based on the sequence of treatments generated using this method, treatment groups (A and B) were written on pieces of cardboard paper and put into sealed opaque envelopes. Each of the opaque envelopes had a serial number on it.

At the ANC, two trained research assistants supervised the randomisation procedure. On each clinic day, all women who consented and met the inclusion criteria were given serial numbers based on their arrival time. Each subject opened the opaque envelope in the presence of a research assistant who recorded the assigned treatment group on the woman's case file. The research assistants monitored and ensured that each woman had a treatment group not influenced by any of the health care providers or member of research team. The trial period was from November 2006 until 30 March 2007.

In the labour room, the senior registrar or consultant confirmed the established labour. In order to prevent bias, no member of the research team participated in this decision. Thereafter, the accompanying companions were provided with an information leaflet that explained their responsibilities as a labour support person. These included: gentle massage of the woman's back during contractions, reassuring words, spiritual support in the form of prayers and also acting as intermediary between the woman and the health care team. After studying the leaflets, they were allowed to seek clarification. The information leaflet was also interpreted for those who were not literate. The attending midwife ensured companions complied with the assigned duties. In addition, the companions were told to offer continuous support (i.e. they were not supposed to leave the side of patients except for eating and using the toilet). The companions stayed until two hours after the birth. The decision for any intervention was based on the judgment of at least two senior clinicians (senior residents or consultant) to minimise bias.

The primary outcome measure was caesarean section rate. Other outcomes were duration of active phase of labour, pain score, need for analgesia, need for oxytocin, time between delivery and initiation of breastfeeding and the emotional experience during labour. The time of initiation to breastfeeding was defined as the interval from childbirth to the point when the child first suckled the breast. The time was recorded by the attending midwife and this was confirmed by a research assistant.

### Nature of data and analysis methods for the breastfeeding study

The data extracted for use in this study consisted of primigravidae in each arm of the study group. Of the total, 94 and 115 primigravid women respectively in the experimental and control groups were extracted for the present study (Figure 1). Women who had operative deliveries were excluded because of varying anesthetic methods that could confound the timing of breastfeeding initiation [13].

### Data analysis

The SPSS version 12.0 statistical software was used for data entry and analysis. Baseline differences between groups were tested using the Chi square and t tests. The median time to initiation of breastfeeding was compared between the two groups using the log rank test. The Cox regression analysis was used to adjust for baseline differences between the groups. The event 'initiation of breastfeeding' was defined as the hazard, hence women who had not initiated breastfeeding at time 't' were assumed to have survived up to that time. Hazard ratios for initiation of breastfeeding and ninety five percent confidence intervals were computed. Level of significance was set at 5%.

## Results

Table 1 shows the biosocial characteristics of the participants. They had similar mean ages which were not significantly different. Women in the experimental group were more likely to have higher education, to belong to the Yoruba tribe, to be skilled and Christian (Table 1).

**Table 1:** Characteristics of participants

*Variables*	*Experimental* *n = 94 (%)*	*Control* *n = 115 (%)*	p value
**Age**			
Mean(SD)	28.3(2.9)	27.5(4.7)	0.13
Occupation*			
Unemployed	0 (0.0)	31 (27.0)	<0.01
Unskilled	13 (13.8)	30 (26.0)	
Skilled	81 (86.2)	54 (47.0)	
Educational status*			
Primary or lower	4 (4.3)	27 (23.5)	<0.01
Secondary and above	90 (95.7)	88 (76.5)	
Religion*			
Christianity	74 (78.7)	72 (62.6)	0.01
Islam	20 (21.3)	43 (37.4)	
Tribe*			
Yoruba	81 (86.2)	73 (63.5)	<0.01
Others	13 (13.8)	42 (36.5)	

*Significant at 5% level and included as covariates in the Cox regression model

**Figure 2:**
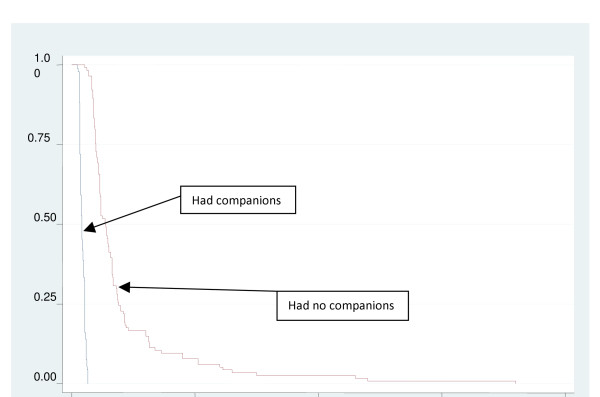
**Cumulative proportions of women yet to initiate breastfeeding between the two groups of women represented in survival curves**.

The number of women in the experimental group who had initiated breastfeeding at 10, 15 and 30 minutes were 91 (97%), 49 (52%) and none respectively. All women in this group had initiated breastfeeding by 26 minutes. However in the control group, none had initiated breastfeeding at 15 minutes and 106 (92%) had not started at 30 minutes. At 1 and 2 hours postpartum, 47 (41%) and 17 women (15%) respectively were yet to initiate breastfeeding, while all had initiated by 12 hours (Figure 2).

The Cox regression analysis was adjusted for baseline differences in the women's socioeconomic characteristics. The analysis showed that women in the experimental group had a hazard ratio of 207.8 (95%CI 49.2, 878.0) of initiating breastfeeding. This indicates that women with companions during labour were much more likely to commence breastfeeding earlier than women without companions (Table 2).

**Table 2:** Hazard ratios from Cox regression of time to initiation of breastfeeding

*Variable*	*Hazard ratio*	*95% Confidence Interval*	p value
**Presence of companion**	207.8	49.2, 878.0	<0.01
**Occupation** (Unskilled vs. skilled)	1.1	0.7, 1.6	0.74
**Education** (Lower vs. Higher)	1.2	0.7, 1.9	0.56
**Tribe** (Yoruba vs. others)	1.2	0.8, 1.9	0.39
**Religion** (Christians vs. Muslims)	1.1	0.8, 1.7	0.45

Defining onset of breastfeeding as the event of interest, survival analysis techniques were used to test the significance of the differences of breastfeeding initiation time between the two groups of women using the log rank test to compare survival times i.e. time to commence breastfeeding. Women in the companion group had time to initiation ranging from 1 to 21 minutes while the control group had times between 26 and 720 minutes. The companion group had a significantly lower median time to initiation of breastfeeding compared to the control group (p < 0.0001) (Table 3, Figure 2).

**Table 3:** Log rank test comparing times to initiation of breastfeeding between primiparous women with a companion during labour and women receiving standard care

Group (n)	Median time to breastfeeding in minutes (range)	95% CI for median time	Log rank statistic	p value
Experimental group (94) Control group (115)	16 (0-26) 54 (21-720)	(14.6, 17.5) (46.6, 61.4)	255.68	<0.01

## Discussion

Time to initiation of breastfeeding is one of the key factors that have consistent influence on the overall breastfeeding practices irrespective of socio-cultural settings [20]. The policy in many health care facilities is for the midwife or any other available skilled providers to encourage and assist in the process of achieving earlier initiation as defined by BFI target. However, as simple as this task sounds, it has been shown that the target of breastfeeding initiation within half an hour were not realizable for a range of reasons [21]. One peculiarity of developing countries, especially Nigeria, is the challenge of disproportionate smaller population of skilled birth attendants compared to women who give birth at the facility. This scenario forms a major cause of delay in breastfeeding initiation especially amongst primiparous women.

In this study, there was a significantly shorter time to breastfeeding initiation in women who had companions compared to those without companions. The observed shorter time may be due to words of encouragement and assistance offered in proper positioning of the baby for ease of breastfeeding by the companions while the woman is still experiencing some after-pains. This finding is similar to previous reports elsewhere. However, this outcome forms the first effort within the Nigerian community that proactively involved family members in the process of breastfeeding initiation within a healthcare facility. The willingness of companions irrespective of gender signifies the importance the community attaches to breastfeeding. It is therefore probable that these companions especially females could be instructed about breastfeeding and can be enlisted as volunteers within their communities by promoting the culture of early initiation, dispelling any superstitions as well as assisting others who may not have delivered at a healthcare facility. This practice may in the long term improve the low level of exclusive breastfeeding and possibly child-spacing practices thereby engendering better maternal and neonatal health within the country. It is important to note that the sociocultural diversity within the country may necessitate modifications to fit into the peculiarities of other regions.

The survival analysis on minute by minute proportions of women who were yet to initiate breastfeeding revealed that all of the women with companions had initiated breastfeeding by the twenty-fifth minute after childbirth. However, the last group among the controls commenced breastfeeding at 720 minutes (12 hours postpartum). One possible challenge of delayed breastfeeding initiation among first time mothers is the likelihood of frustration and failure to cope with the perceived stress/anxiety of the expected responsibility, leading to a vicious cycle. This attitude has been reported to prevent mothers from practicing exclusive breastfeeding in the long term. On the other hand, the positive results observed among those with support persons suggests the possibility of using companions to complement the services of health care providers in busy maternity homes or primary health centres, which lack enough manpower to supervise early commencement of breastfeeding. Adoption of this concept into the Nigerian maternity health care system will further augment the attainment of BFI across the country at no cost.

There is the possibility that differing biosocial variables such as occupation, tribe, religion and mode of delivery between the study groups influenced the findings. However, Cox regression analysis was performed to eliminate these potential confounding factors. The results indicated that only the presence of a companion during labour had a significant positive influence on the time to breastfeeding initiation. It can then be argued that offering support during the process of childbirth was associated with better outcomes as none of the support persons attended antenatal health talks on breastfeeding issues and a significant proportion were their male partners. The outcomes based on different types of companions brought by each woman were not explored and this will require further research in future in order to be able to determine whether there is any effect.

In spite of the observed benefit of earlier breastfeeding initiation among the experimental group in this data set, the extrapolation of the findings are limited by several factors: the failure of the randomization process of the entire data, and the non-blinding of the midwives to the treatment allocation. Furthermore, lack of follow-up of these women is another potential limitation and could limit the extrapolation of our findings. Lastly, our study population is mostly drawn from middle and high social class and this may not necessarily reflect the total spectrum of women in Nigeria. However, breastfeeding is still the norm in most communities within the country except women with clear contraindication.

## Conclusion

Use of companions during childbirth of first-time Nigerian mothers is associated with early breastfeeding initiation. This strategy could potentially promote positive attitudes towards BFI policy in spite of the inadequate postpartum care services.

## Competing interests

The authors declare that they have no competing interests.

## Authors' contributions

All the authors participated in all the stages of the research from conceptual framework, proposal writing, conduct of the study and eventual write-up of the manuscript. Specifically, IOMB and OAO conducted relevant literature searches and proposal writing, recruitment of subjects and writing of the manuscript while methodology design including randomisation, quality assurance and data analysis were performed by BOA.
